# Enhancing Robustness of Machine Learning Integration With Routine Laboratory Blood Tests to Predict Inpatient Mortality After Intracerebral Hemorrhage

**DOI:** 10.3389/fneur.2021.790682

**Published:** 2022-01-03

**Authors:** Wei Chen, Xiangkui Li, Lu Ma, Dong Li

**Affiliations:** ^1^Department of Neurosurgery, West China Hospital of Sichuan University, Chengdu, China; ^2^West China Biomedical Big Data Center, West China Hospital of Sichuan University, Chengdu, China; ^3^Med-X Center for Informatics, Sichuan University, Chengdu, China; ^4^Division of Hospital Medicine, Emory School of Medicine, Atlanta, GA, United States

**Keywords:** machine learning, intracerebral hemorrhage, laboratory profiles, prediction, prognostication

## Abstract

**Objective:** The accurate evaluation of outcomes at a personalized level in patients with intracerebral hemorrhage (ICH) is critical clinical implications. This study aims to evaluate how machine learning integrates with routine laboratory tests and electronic health records (EHRs) data to predict inpatient mortality after ICH.

**Methods:** In this machine learning-based prognostic study, we included 1,835 consecutive patients with acute ICH between October 2010 and December 2018. The model building process incorporated five pre-implant ICH score variables (clinical features) and 13 out of 59 available routine laboratory parameters. We assessed model performance according to a range of learning metrics, such as the mean area under the receiver operating characteristic curve [AUROC]. We also used the Shapley additive explanation algorithm to explain the prediction model.

**Results:** Machine learning models using laboratory data achieved AUROCs of 0.71–0.82 in a split-by-year development/testing scheme. The non-linear eXtreme Gradient Boosting model yielded the highest prediction accuracy. In the held-out validation set of development cohort, the predictive model using comprehensive clinical and laboratory parameters outperformed those using clinical alone in predicting in-hospital mortality (AUROC [95% bootstrap confidence interval], 0.899 [0.897–0.901] vs. 0.875 [0.872–0.877]; *P* <0.001), with over 81% accuracy, sensitivity, and specificity. We observed similar performance in the testing set.

**Conclusions:** Machine learning integrated with routine laboratory tests and EHRs could significantly promote the accuracy of inpatient ICH mortality prediction. This multidimensional composite prediction strategy might become an intelligent assistive prediction for ICH risk reclassification and offer an example for precision medicine.

## Introduction

To date, spontaneous intracerebral hemorrhage (ICH), a leading cause of stroke and a life-threatening and disabling illness, remains a severe condition worldwide (1–4). Early aggressive care draws more advocates for spontaneous ICH outlined by the current practice guideline, requiring early and accurate identification of individuals who are at risk for unfavorable outcomes (5). In response to such urgent needs, robust risk estimators are highly recommended. The ICH score (6) is a classic prediction tool widely used in ICH management currently. However, this score assessment system comprises five risk factors: the Glasgow Coma Scale (GCS) score, ICH volume, intraventricular hemorrhage (IVH), the infratentorial origin of ICH, and age simple to use in clinical practice. However, such a clinical grading scale is used as a stand-alone risk assessment system and less integrated with additional dimension information, such as routine clinical laboratory profiles, for more comprehensive and precise individual risk assessment.

Integrating routine laboratory blood tests for risk predictions in patients with ICH may have several important implications. Firstly, linking laboratory biomarkers to augment traditional ICH risk prediction could benefit individualized disease management. Those laboratory indicators provide additional multidimensional information, which is hardly captured by static electronic health records (EHRs) and even images from the PACS (picture archiving and communication systems) (7). Secondly, the multiple laboratory blood tests for inpatients provided dynamic clues for the progression and pathological changes of the disease over time (8, 9). In addition, using those objectively measured and readily available data would allow cost-effective assessment and intervention of ICH without posing an additional burden in an urgent clinical scenario. However, most conventional tests are limers, making clinical replication difficult. Besides, traditional analytical methods are difficult to adapt to large quantities of measurements.

Machine learning (ML) is a promising strategy for learning complex rules and objectively synthesizing and interpreting the patterns from multidimensional datasets (10, 11). ML could incorporate an extensive array of predictors in a non-linear pattern and use multiple interactions to enhance prediction accuracy (10–13). In recent years, ML has been used for prediction and decision-making in a multitude of ICH (14–19). Unfortunately, to our knowledge, there has been no effort to use ML to take advantage of blood laboratory data to help physicians predict outcomes at a personalized level in patients with ICH who undergo assessments during routine clinical care. Moreover, the black box-like feature of these algorithms that usually limits their usefulness in clinical practice (20, 21). Clinicians are more likely to trust and use ML methods when they are explainable. In doing so, we introduced the SHapley Additive exPlanation (SHAP) algorithm to help explain the prediction model (22, 23). With the aforementioned considerations in mind, we initially introduced the SHapley Additive exPlanation (SHAP) algorithm to enhance the robustness of ML integrated with routine laboratory blood tests to predict inpatient mortality after ICH.

## Materials and Methods

### Study Population

The patients from the West China Hospital of Sichuan University, tertiary care, academic and non-profit hospital with 4,300 beds, had ~279,000 patients discharged in 2019 (24). Consecutive patients with acute spontaneous ICH admitted to the hospital between October 1, 2010, and December 31, 2018, were screened. The inclusion criteria include (1) ≥18 years old, (2) with the first-ever diagnosis of spontaneous ICH within 24 h that was confirmed by the head CT scan, (3) laboratory blood tests are available at admission, and (4) complete discharge diagnosis and outcomes records are available. We excluded patients with primary intraventricular hemorrhage (IVH) and secondary ICH, such as trauma, tumors, or vascular structural abnormalities (e.g., aneurysms and arteriovenous malformation). The West China Hospital of Sichuan University Biomedical Research Ethics Committee reviewed and waived informed consent (No. 20-1209) due to a retrospective data analysis characterized by desensitized data. This study adheres to the Transparent Reporting of a Multivariable Prediction Model for Individual Prognosis or Diagnosis (TRIPOD) reporting guideline for diagnostic and prognostic studies.

### Data Source

Clinical data were derived from the EHRs system. Demographic information and clinical characteristics were retrieved, including age, sex, time from the symptom onset to admission (hours), length of stay (days), admission GCS score, lifestyle risk factors (smoking and alcohol use), recorded comorbidities (hypertension and diabetes mellitus), and neurosurgical operation (hematoma evacuation). Hypertension and diabetes mellitus was defined as diagnosed or documented—all blood laboratory biomarkers obtained from standard laboratory tests in routine clinical practice at the time of admission. Of note, only measures that were routinely available for most patients were included. As a result, a total of 59 laboratory blood parameters were included in the present analysis (see details in the Supplementary Table 1). Neuroimaging data on hematoma volume [mL; calculated using the formula ABC/2 (25)], hematoma locations (infratentorial origin or not), and the presence of an IVH were also collected manually. The primary outcome of interest was all-cause in-hospital mortality retrieved from paramedic EHRs. The outcome label of in-hospital mortality was defined as a discharge disposition of “expired.”

If data elements were not structured, they were manually processed by two experienced investigators (X.L. and a non-author) who were blinded to the study's aims. Missing values for each variable were assigned a default value of “NA” (not available), which served as an indicator for missingness. This allowed the algorithm to include observations with missing features and to gain signals from missingness itself. Prior studies have also shown that the eXtreme Gradient Boosting (XGBoost) model can gain signals from missingness (or non-missingness) without resorting to imputation techniques (26).

### ML Algorithms and Model Selection

The dataset was separated in a split-by-year training/test scheme. We trained a ML model on data from patients who were admitted to the hospital from October 1, 2010 to December 31, 2016. We randomly selected 90% of patients for model training in the training dataset and a held-out 10% for validation. We used the annotated 59 laboratory biomarkers to train and evaluate several supervised learning algorithms and compare their performances. We experimented with four classifiers: logistic regression, classification and regression trees, random forest, and the XGBoost model. Initial tests demonstrated the superior performance of the XGBoost model compared with the other three models, so we selected this model using the Python programming language (Python Software Foundation) with the XGBoost package as our preferred model, with the 13 most influential circulating parameters for the exciting outcome chosen using five cross-validations. We created a base model using the 5 ICH risk score variables. We then provided additional performance characteristics for the selected approach to focus on the differences between ML with only clinical data (ML-clinical) and ML with clinical and laboratory data (ML-combined). In the test dataset, the model was further validated in an additional cohort spanning from January 1, 2017 to December 31, 2018. The SHAP value was used to illustrate the positive or negative effects of the 18 features attributed to the XGBoost model. We also used the SHAP dependence plot to explain how a single feature of these laboratory biomarkers affects the output of the XGBoost prediction model.

### Statistical Analysis

Continuous variables are presented as mean ± standard deviation (SD) or medians with interquartile ranges (IQR). Categorical variables are presented as numbers with percentages. As appropriate, comparisons of intergroup differences were analyzed using the Student *t*-test, Mann-Whitney *U*-test, or Chi-square test. The performances of the ML models were evaluated by receiver operating characteristic (ROC) curve, Kaplan–Meier curve, confusion matrix metrics, and evaluation metrics, including area under the ROC curve (AUROC), precision, sensitivity, specificity, and accuracy. The formulas for computing the metrics were described elsewhere. We calculated 95% confidence intervals (CIs) for comparisons of AUROC using 1,000 bootstrap replications. Probabilities of more than 2/3 quantile were assigned to high-risk and otherwise to low risk. All statistical analyses were performed using R, version 3.3 (R Foundation for Statistical Computing) and Python, version 2.7 (Python Software Foundation) software. A 2- tailed *P* <0.05 was considered statistical significance.

## Results

### Study Cohort and Population

**Figure 1:**
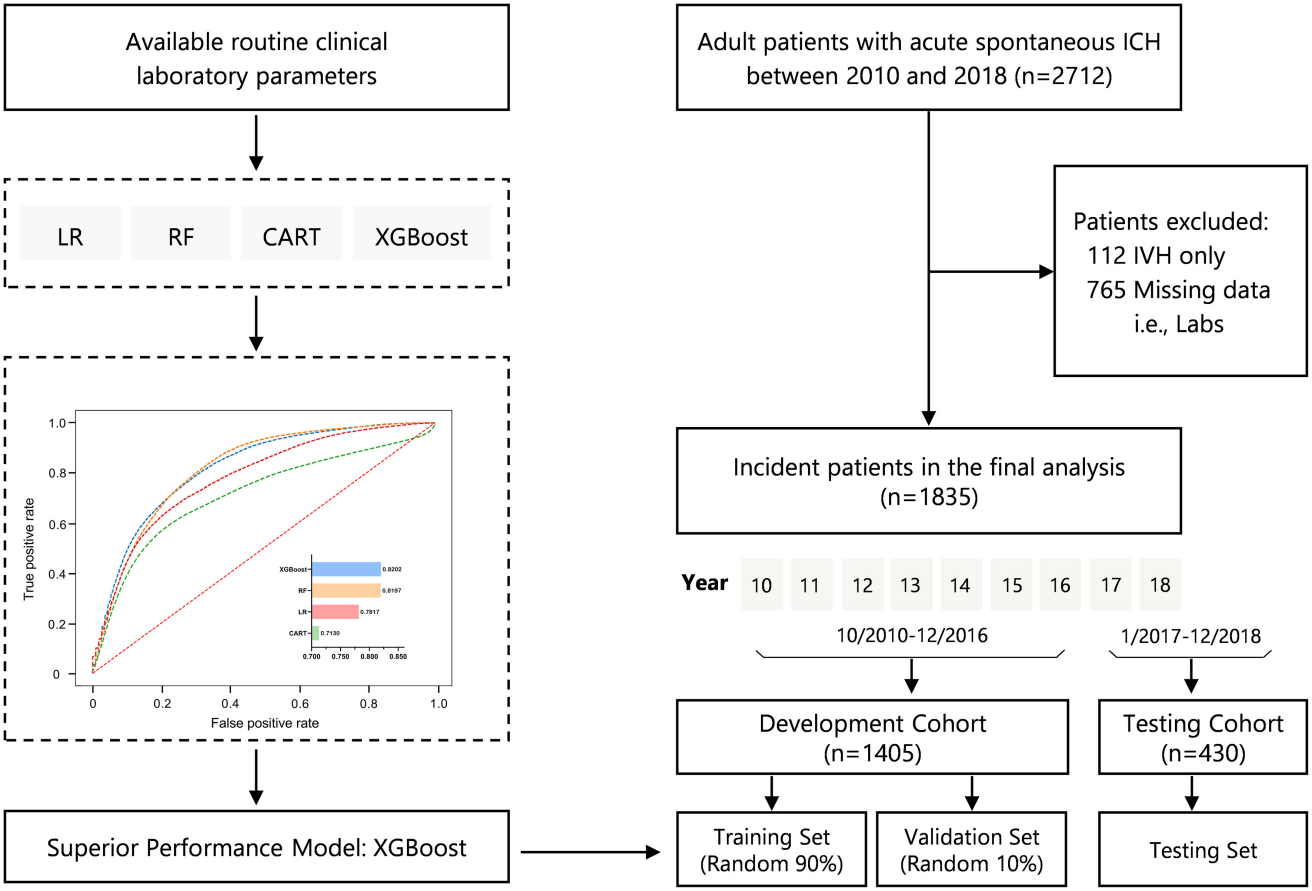
Study design and study flowchart. CART, classification and regression trees; ICH, intracerebral hemorrhage; IVH, intraventricular hemorrhage; LR, logistic regression; RF, random forest; XGBoost, eXtreme Gradient Boosting.

Figure 1 illustrates the study design and study flow chart. We included a total of 1,835 patients with ICH in the present analysis; 1,405 of these patients were included in the development cohort (training and held-out validation set), and 430 were used as the testing cohort. The mean age was 59 ± 15 years, and 67.9% were men. The overall percentages of all-cause in-hospital mortality for the full population, development, and testing cohorts were 20.3, 19.4, and 23.0%, respectively.

Table 1 summarizes the baseline characteristics of the study population. Patient's characteristics were similar between the development and testing cohort except for GCS score (82 of 430 patients with GCS score 3–4 in the development cohort [19.1%] vs. 188 of 1,405 patients in the testing cohort [13.4%]; *P* = 0.013), the lifestyle risk factor of current smoking (*P* = 0.043), and undergoing hematoma evacuation therapy (*P* = 0.045) (Table 1). Patients who died were older, had a shorter time from the symptom onset to admission, and more temporary hospital stay, to have a comorbidity of diabetes mellitus. In addition, they were more likely to have lower GCS scores and larger ICH volume, had hematoma located in infratentorial, accompanied by a higher presence of IVH. At the same time, they were less likely to have undergone hematoma evacuation therapy (all *P* < 0.05), as presented in Table 1.

**Table 1:** Demographic and baseline characteristics of study patients.

**Characteristics**	**Overall**		**Dataset stratification**			**Outcomes**		
	**(*N* = 1,835)**	**% missing**	**Development dataset (*n* = 1,405)**	**Testing dataset (*n* = 430)**	***P*-value**	**Discharged (*n* = 1,463)**	**Death (*n* = 372)**	***P*-value**
Age^*^								
Mean, years	59 ± 15	0	59 ± 15	60 ± 15	0.125	59 ± 14	62 ± 15	<0.001
Distribution					0.172			<0.001
≥80	162 (8.8%)	0	117 (8.3%)	45 (10.5%)		110 (7.5%)	52 (14.0%)	
<80	1,673 (91.2%)	0	1,288 (91.7%)	385 (89.5%)		1,353 (92.5%)	320 (86.0%)	
Sex								
Female	589 (32.1%)	0	438 (31.2%)	151 (35.1%)	0.126	469 (32.1%)	120 (32.3%)	0.941
Male	1,246 (67.9%)	0	967 (68.8%)	279 (64.9%)		994 (67.9%)	252 (67.7%)	
Time from symptom onset to admission, hours	5.0 (4.0–9.0)	0	6.0 (4.0–9.0)	4.0 (3.0–7.0)	0.288	6.0 (4.0–9.0)	4.0 (3.0–7.0)	<0.001
Length of stay, days	5.0 (2.0–9.0)	0	5.0 (2.0–9.0)	5.0 (2.0–9.0)	0.143	6.0 (4.0–10.0)	1.0 (1.0–5.0)	<0.001
Lifestyle risk factors								
Current smoking	532 (29.0%)	0	424 (30.2%)	108 (25.1%)	0.043	421 (28.8%)	111 (29.8%)	0.687
Alcohol use	263 (14.3%)	0	204 (14.5%)	59 (13.7%)	0.679	205 (14.0%)	58 (15.6%)	0.438
Comorbidities								
Hypertension	1,367 (74.5%)	0	1,050 (74.7%)	317 (73.7%)	0.674	1,104 (75.5%)	263 (70.7%)	0.060
Diabetes mellitus	168 (9.2%)	0	125 (8.9%)	43 (10.0%)	0.488	119 (8.1%)	49 (13.2%)	0.003
Hematoma evacuation	99 (5.4%)	0	84 (6.0%)	15 (3.5%)	0.045	90 (6.2%)	9 (2.4%)	0.004
GCS score^*^					0.013			<0.001
3–4	270 (14.7%)	0	188 (13.4%)	82 (19.1%)		82 (5.6%)	188 (50.5%)	
5–12	437 (23.8%)	0	343 (24.4%)	94 (21.9%)		312 (21.3%)	125 (33.6%)	
13–15	1,128 (61.5%)	0	874 (62.2%)	254 (59.1%)		1,069 (73.1%)	59 (15.9%)	
ICH volume^*^								
Median, mL	15.1 (6.3–36.5)	4.5%	14.9 (6.4–35.4)	15.7 (6.2–38.9)	0.474	12.6 (5.6–27.2)	46.4 (18.5–92.1)	<0.001
Distribution					0.451			<0.001
≥30	527 (30.1%)	4.5%	397 (29.6%)	130 (31.6%)		308 (22.0%)	219 (61.9%)	
<30	1,226 (69.9%)	4.5%	944 (70.4%)	282 (68.4%)		1,091 (78.0%)	135 (38.1%)	
Presence of IVH^*^					0.340			<0.001
Yes	488 (26.6%)	0	366 (26.0%)	122 (28.4%)		354 (24.2%)	134 (36.0%)	
No	1,347 (73.4%)	0	1,039 (74.0%)	308 (71.6%)		1,109 (75.8%)	238 (64.0%)	
Infratentorial origin of ICH^*^					0.146			<0.001
Yes	455 (24.8%)	0	337 (24.0%)	118 (27.4%)		328 (22.4%)	127 (34.1%)	
No	1,380 (75.2%)	0	337 (24.0%)	118 (27.4%)		1,135 (77.6%)	245 (65.9%)	

*Data are presented as mean ± SD or median (interquartile range) and n (%). ICH, intracerebral hemorrhage; IVH, intraventricular hemorrhage; GCS, Glasgow Coma Scale*.

* *Those variables included in the ICH score*.

### Model Performance and Comparisons

**Figure 2:**
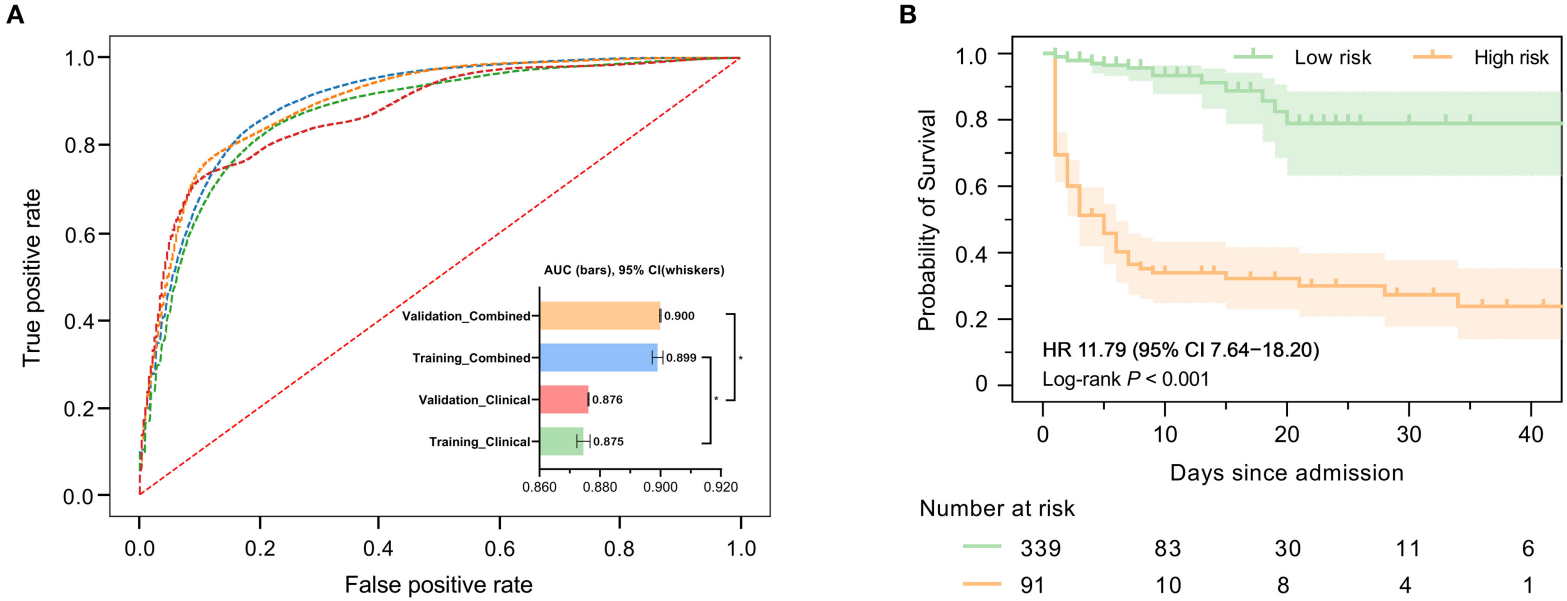
Predictive performance of machine learning-based models. **(A)** Areas under the curve (AUCs) to assess the performance of in-hospital mortality risk prediction of models in the development cohort (held-out for validation) and testing cohort using clinical features only or combined with selected laboratory variables. The AUC for ML combined with clinical features and laboratory variables was significantly higher than that used clinical features only. **(B)** Kaplan–Meier curves indicate the overall survival of patients with high and low mortality risk in the validation cohort. The tick marks refer to censored patients. The dark green or orange line indicates the survival probability, and the light green or orange areas represent the 95% confidence interval of survival probability (log-rank *P* < 0.001).

Figure 2A demonstrates the model diagnostic performance in terms of AUROC in the validation and testing sets. Overall, ML models using laboratory data achieved AUROCs of 0.71–0.82 in a split-by-year development/testing scheme, with the non-linear XGBoost model yielded the highest prediction accuracy. In the held-out validation set, the predictive model using comprehensive clinical and laboratory parameters outperformed those using clinical alone in predicting in-hospital mortality (AUROC [95% bootstrap CI], 0.899 [0.897–0.901] vs. 0.875, [0.872–0.877]; *P* < 0.001). Similar performance was observed in the testing set. Table 2 presents the classification model performance when applied to the validation set and testing set. Interestingly, the performance in the held-out validation cohort was worse than the testing cohort in terms of precision (77.5 vs. 81.3%), sensibility (77.7 vs. 81.2%), specificity (77.7 vs. 81.2%), and accuracy (85.8 vs. 86.7%). Additional statistical measures of the performance of models are reported in the Supplementary Table 2.

**Table 2:** Statistical measures of the performance of models^*^.

	**Precision**	**Sensitivity**	**Specificity**	**Accuracy**
Development cohort (held-out validation)	0.775 (0.772–0.778)	0.777 (0.774–0.779)	0.777 (0.774–0.779)	0.858 (0.857–0.860)
Testing cohort	0.813 (0.812–0.813)	0.812 (0.811–0.812)	0.812 (0.811–0.812)	0.867 (0.867–0.867)

* *The precision, sensibility, specificity, and accuracy were presented by mean (95% CI)*.

We further investigated precise individual-level mortality risk. With the time from admission to death or discharge as the endpoint, Kaplan-Meier analysis further confirmed that the ML model could robustly stratify patients by mortality risk. High-risk patients labeled by our model were significantly less likely to survive than low-risk patients in the testing cohort with a hazard ratio of 11.79 (95% CI: 7.64–18.20), highlighting the capability of the model to accurately predict the prognosis of ICH patients, as shown in Figure 2B.

### Most Influential Predictors

**Figure 3:**
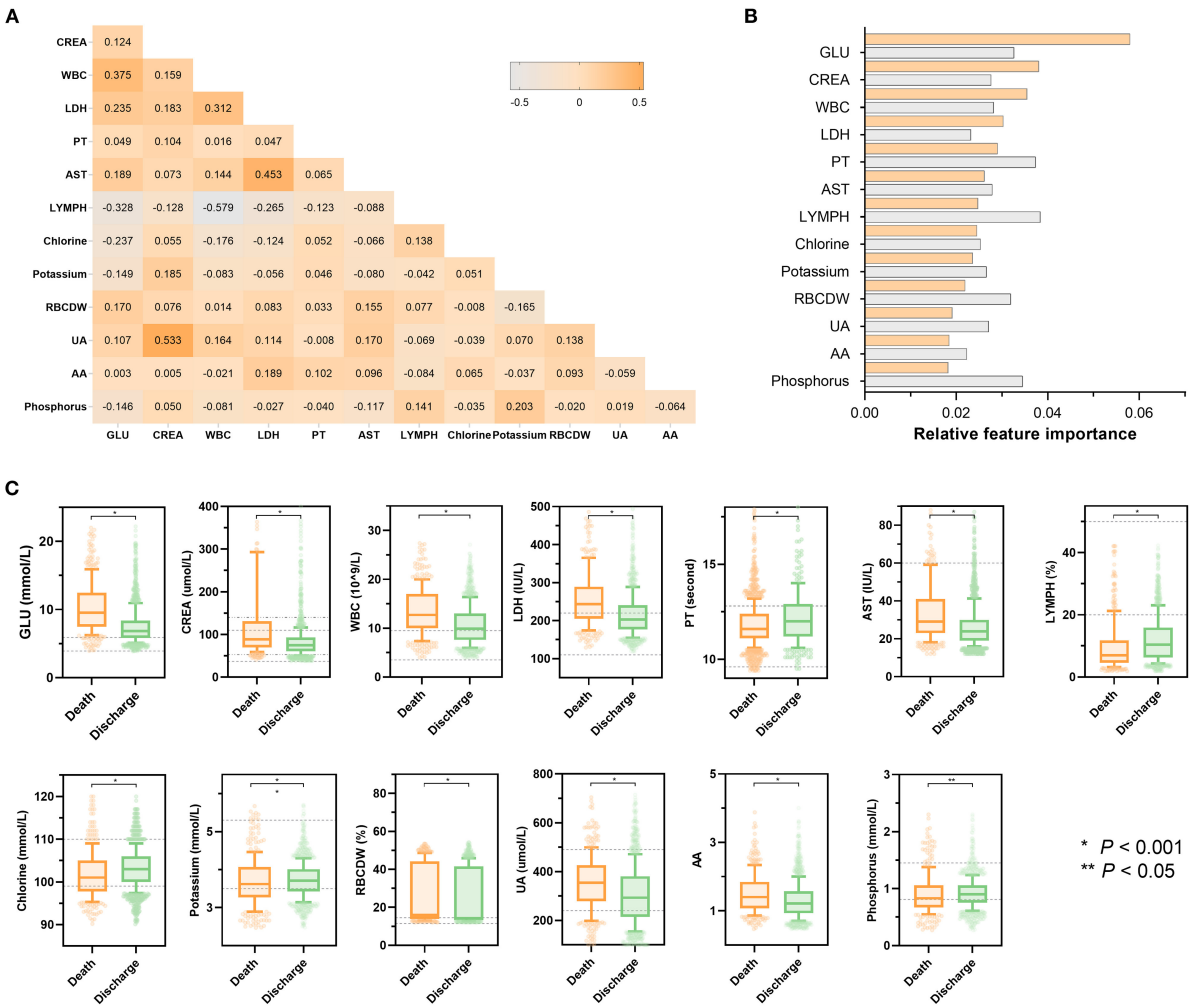
Statistical analysis of features included in models. **(A)** Heatmap represents the correlation between all laboratory features included in the model using Spearman's correlation coefficient. The colors in the plot represent the correlation coefficients. The numbers in the triangle represent the value of the correlation coefficient. **(B)** Scaled importance rank of all laboratory features included in the models. The size of bars represents the value of relative importance. The bars with orange represent the feature included in the model that did not contain clinical features, while the gray bar represents the feature included in the model that did not contain clinical features. **(C)** Box and jitter plots show the distribution of all laboratory features included in the present study between deceased patients (*n* = 373) and discharged patients (*n* = 1,466). The centerline represents the median of the feature. Box limits represent upper and lower quartiles. Whiskers represent 1.5 times the interquartile range. Color points represent outliers. Mann-Whitney *U*-test was used in the univariate comparison between groups, and a two-tailed *P* < 0.05 was considered statistically significant. AA, the ratio of alanine aminotransferase to AST; AST, aspartate aminotransferase; HR, hazard ratio; CI, confidence interval; CREA, creatinine; GLU, blood glucose; LDH, low-density lipoprotein; LYMPH, percentage of lymphocytes; PT, prothrombin time; RBCDW, red blood cell distribution width (CV); UA, uric acid; WBC, white blood cell count.

Figure 3 presents the statistical analysis of the essential predictors selected by the best classifier (non-linear XGBoost model) from 59 laboratory blood parameters in the training set. A total of 13 features were eventually chosen for modeling, with each variable included in the model has been varying importance over in-hospital mortality. Spearman's correlation coefficient analysis using raw data of the 13 features exhibited various degrees of correlation (Figure 3A). Among the top 3 of those selected variables, blood glucose was selected as the top-most influential predictor in the model, followed by creatinine, and white blood cell count, respectively (Figure 3B). In the model that includes both laboratory blood parameters and clinical variables, the relative importance of those included parameters on the model's predictions was observed to have altered mildly or moderately. Standard box plots presented the included parameters' distributions between discharged and deceased patients (Figure 3C). According to datasets and outcomes, additional distributions of these laboratory values can be found in Supplementary Tables 3, 4.

### Model Explanation

**Figure 4:**
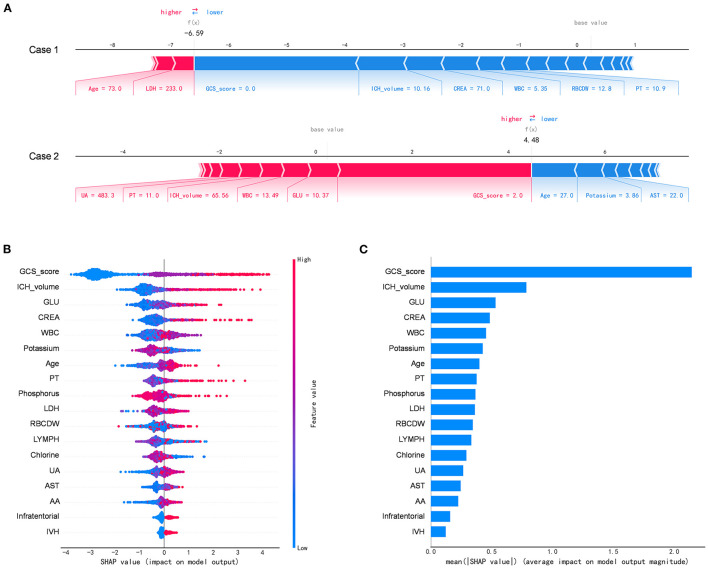
The Shapley additive explanations algorithm for the machine learning model. **(A)** Illustration of the impact of features for two patient-specific predictions using the Shapley additive explanations (SHAP) algorithm. Case 1 was alive, while case 2 was deceased during the hospitalization. The factors positively (red) and negatively (blue) impacting the construction of the model, with the size of the bars depicting importance. For example, Glasgow Coma Scale score 3–4 (=2 points) is an essential variable increase the risk of in-hospital mortality in case 2. **(B)** The attributes of the features in the black-box model. Each line represents a feature, and the abscissa is the SHAP value, which represents the degree of influence on the outcome. Each dot represents a sample. The redder the color, the greater the value of the feature, and the bluer the color, the lower the value. **(C)** Ranking of feature importance indicated by SHAP. The most critical variable has the highest mean of absolute SHAP values. AA, the ratio of alanine aminotransferase to AST; AST, aspartate aminotransferase; CREA, creatinine; GLU, blood glucose; LDH, low-density lipoprotein; LYMPH, percentage of lymphocytes; PT, prothrombin time; RBCDW, red blood cell distribution width (CV); UA, uric acid; WBC, white blood cell count.

Figure 4 depicts the SHAP algorithm for the interpretations of the ML model. We illustrated two representative cases from the holdout validation set (Figure 4A). In this example, the patient's GCS scores 3–4 (=2 points) is the most crucial variable to increase the risk of in-hospital mortality in case 1 who died, while GCS scores 13–15 (=0) is the most critical risk-decreasing variable in case 2 who discharged. Additionally, the contribution of each of the 13 features in the model was visualized by applying the SHAP summary plot (Figure 4B). This allowed understanding how a single variable influences the output of the XGBoost prediction model. According to the prediction model, the higher the SHAP value of a feature, the more likely death becomes. Furthermore, we used the SHAP algorithm to rank the importance of all the included variables for the prediction model (Figure 4C). The most crucial variable has the highest mean of absolute SHAP values.

## Discussion

To our knowledge, this study represents the first large-scale report leveraging explainable ML algorithms to generate accurate outcome predictions for the ICH population by combining clinical and routine laboratory blood test data. The non-linear XGBoost model could automatically derive critical variables from comprehensive laboratory results such as glucose, creatinine, and white blood cell count. This pipeline analysis model is suitable for integrating clinical data with readily available laboratory profile results for dynamic disease progression prediction. Our results reveal that an ML-based algorithm offers a great potential to enhance accuracy in predicting personalized in-hospital all-cause mortality in patients with ICH. These findings add insights into ML algorithms designed to improve optimal evaluation and clinical decisions in digital health care.

Currently, the ability to optimally assess risk in individual patients remains a significant challenge in ICH. Such a challenge is mainly because the evaluation of such emergent conditions is often time pressing. It is crucial but difficult for clinicians to make rapid and accurate clinical decisions in a short period when facing a broad array of available information. Traditional clinical practice has dealt with this situation, in part, by using various risk prediction scoring systems, some of which are summarized by Gregório et al. (27). However, these scoring systems are limited by the amount of clinically available measurements, particularly laboratory measures. The presented ML strategy provides insight into integrating algorithm (software) with routine laboratory tests (facilities and other resources) and interpretation beyond what is provided by conventional statistics. More importantly, our study introduced a novel unsupervised learning algorithm strategy to derive labs without a priori assumptions about the influence or weighting of individual factors or how they may interact. This approach allowed us to filter through the massive laboratory parameters to identify potential risk factors for death after ICH.

As a result, certain variables linked to patient survival were ultimately included in the prediction model. For example, in our model, blood glucose derived from labs is the most important feature for predicting ICH mortality during hospitalization, as considerable previous studies have supported this finding (28–30). Some of which, including creatinine (31), white blood cell count (32), prothrombin time (33), chlorine (34), potassium (35), percentage of lymphocytes (36), red blood cell distribution width (37), uric acid (38), and phosphorus (39) also correspond with previously investigated risk factors from previous clinical studies. These features were presented in nearly two-thirds of the overall selecting variables (8 out of 13), suggesting our model is reliable. Notably, our model identified several biomarkers that other ICH studies have not reported, such as the aspartate aminotransferase and the ratio of alanine aminotransferase to aspartate aminotransferase.

When integrating those selecting features, the performance of the ML-combined score was superior to the clinical risk metric that is traditionally used to study prognostic outcomes after ICH. Several studies have been published using ML to predict outcomes (primarily survival and function) in patients with ICH (15–19). The reported performances (AUROC) vary from 0.63 to 0.92, mostly around 0.75–0.85. Therefore, our model performance is consistent with these studies but with enhancing clinical utility and generalizability. We developed a pipeline automatic screening model that is especially suitable for large-scale clinical data analysis with comprehensive laboratory parameters in urgent situations to determine disease progression and conditions. In addition, our model was able to provide a relatively high AUROC and predicted a probability of death for each patient. At the point of care, the estimated probabilities can be used to construct risk strata to quickly separate low-risk patients from those with a high risk of mortality. This technique could be precious for determining who is most likely to benefit when limited resources and assessment time is pressing. Those results further highlight ML technology can assist physicians with digesting a large amount of information and be critical to fully utilizing these growing datasets to help transform and optimize medical practice.

Several limitations of this study need to point out. Firstly, this study relied on retrospective data, and deriving clinical information from EHRs is an inherent limitation (40). For example, some critical variables related to the mortality or functional outcomes of ICH, such as baseline NIHSS and hematoma extension, were not included in the model. Secondly, the laboratory measurements at the time of admission were used in this study. Dynamic laboratory parameters may provide more information to the ML model. Bedsides, to assess the added predictive value of laboratory information concisely, straightforward, and comparably, we only input the variables from classic ICH risk score variables rather than selecting all clinical variables using the ML model. Third, as with any other ML-based study, the model is subject to the constraints of the specific population that it trains on (41). Further validation in a multicenter setting with different patient populations is warranted because our model was performed in a single-center study and not externally validated.

## Conclusion

In conclusion, our study initially provided translational data to support that ML integrated with laboratory blood tests can effectively predict inpatient mortality after intracerebral hemorrhage. The above strategy might become a novel, intelligent clinical companion diagnostic tool for risk assessment of ICH, integrating with the electronic health record and laboratory blood test results.

## Data Availability Statement

The raw data supporting the conclusions of this article will be made available by the authors, without undue reservation.

## Ethics Statement

The studies involving human participants were reviewed and approved by West China Hospital of Sichuan University Biomedical Research Ethics Committee. Written informed consent for participation was not required for this study in accordance with the national legislation and the institutional requirements.

## Author Contributions

The study design was conceived by WC and DL. All authors drafted the article or revised it critically for important intellectual content, approved the final draft to be published, and agree to be accountable for all aspects of the work in ensuring that questions related to the accuracy or integrity of any part of the work are appropriately investigated and resolved.

## Funding

This study was funded by the Sichuan Science and Technology Program (Grant No. 2021YFS0203), the 1·3·5 project for disciplines of excellence–Clinical Research Incubation Project and Post-Doctor Research Project, West China Hospital, Sichuan University (Grant Nos. ZYJC18010 and 2020HXBH156), the Chengdu Science and Technology Bureau (Grant Nos. 2019-YF09-00211-SN and 2021-YF05-01006-SN) and the National key R&D Program of China (Grant Nos. 2018YFA 0108604 and 2018YFA0108603). The funding agency was not involved in the study.

## Conflict of Interest

The authors declare that the research was conducted in the absence of any commercial or financial relationships that could be construed as a potential conflict of interest.

## Publisher's Note

All claims expressed in this article are solely those of the authors and do not necessarily represent those of their affiliated organizations, or those of the publisher, the editors and the reviewers. Any product that may be evaluated in this article, or claim that may be made by its manufacturer, is not guaranteed or endorsed by the publisher.
