# Supplementary material for: Enhancing Robustness of Machine Learning Integration With Routine Laboratory Blood Tests to Predict Inpatient Mortality After Intracerebral Hemorrhage
Source: Front Neurol. 2022 Jan 3;12:790682. doi: 10.3389/fneur.2021.790682 (PMC8761736; doi:10.3389/fneur.2021.790682)
Supplement: Supplementary file 1 [file Data_Sheet_1.PDF]

Supplementary Material

Supplementary Table S1. Overview of all the laboratory variables that was available for modelling.

Variable	Missing (%)
**Blood routine examination**	2.1%
Red blood cell count (RBC)	2.1%
Hemoglobin (HGB)	2.0%
Hematocrit	2.2%
Mean corpuscular volume (MCV)	5.4%
Mean erythrocyte HGB content	5.2%
Mean erythrocyte HGB concentration	2.0%
RBC distribution width_CV (RBCDW)	1.9%
RBC distribution width_SD (RBCDW)	2.2%
Blood platelet count (PLT)	2.2%
White blood cell count (WBC)	2.2%
Percentage of neutrophilic granulocytes (NEUT%)	2.2%
Percentage of lymphocytes (LYMPH%)	1.9%
Percentage of monocytes (MONO%)	2.5%
Percentage of eosinophils (EO%)	35.7%
Percentage of basophils (BASO%)	16.5%
Neutral lobulated granulocytes absolute value (NEUT#)	2.3%
Absolute value of lymphocyte (LYMPH#)	2.2%
Absolute value of monocytes (MONO#)	2.8%
Absolute value of eosinophil (EO#)	35.5%
Absolute value of basophils (BASO#)	16.2%
**Coagulation function test**	
Prothrombin time (PT)	2.7%
Interntionl normlized rtio (INR)	2.8%
Activated partial thromboplastin time (APTT)	2.8%
Activated partial thromboplastin time ratio (APTTR)	3.3%
Thrombin time (TT)	2.9%
Prethrombin time ratio (PTR)	3.0%
Fibrinogen	2.9%
**Blood biochemical index**	
Total bilirubin (TBIL)	2.2%
Direct bilirubin (DBIL)	2.2%
Indirect bilirubin (IBIL)	2.2%
Alanine aminotransferase (ALT)	2.5%

Aspartate aminotransferase (AST)	2.1%
ALT/AST	2.1%
Total protein (TP)	2.2%
Albumin (ALB)	2.3%
Globulin (GLB)	2.2%
Ratio of albumin to globulin	2.2%
Glucose (GLU)	2.2%
Urea (UREA)	2.2%
Creatinine (CREA)	2.2%
Serum cystatin C	2.2%
Uric acid (UA)	2.1%
Triglyceride	7.7%
Cholesterol	6.9%
High density lipoprotein (HDL)	6.9%
Low density lipoprotein (LDL)	6.9%
Alkaline phosphatase (ALP)	2.2%
Glutamyl transpeptidase (GGT)	1.9%
Creatine kinase (CK)	7.2%
Lactic dehydrogenase (LDH)	7.4%
Hydroxybutyrate Dehydrogenase	7.3%
Sodium	2.8%
Potassium	2.8%
Chlorine	2.8%
Carbon dioxide combining power	3.4%
Anion gap	3.4%
Calcium	6.4%
Magnesium	6.9%
Serum inorganic phosphorus (phosphorus)	6.3%

Supplementary Table S2. Distribution of extracted laboratory features with regard to the data sets.

Models	Precision		Recall		F1-score	
	Validation	Test	Validation	Test	Validation	Test
Clinical features only	0.85	0.83	0.82	0.79	0.82	0.80
Combined clinical and biomarker variables	0.88	0.87	0.87	0.87	0.87	0.87

Supplementary Table S3. Association of extracted laboratory features with in-hospital mortality.

Variable	Discharged (n=1463)	Death (n=372)	Standardize Difference* (95% CI)	*P*-value
GLU	6.8 (5.8-8.4)	9.5 (7.5-12.4)	0.8 (0.7, 0.9)	<0.001
CREA	75.0 (61.0-93.0)	89.0 (70.0-130.5)	0.4 (0.3, 0.6)	<0.001
WBC	9.9 (7.6-12.9)	12.7 (10.0-17.0)	0.6 (0.5, 0.7)	<0.001
LDH	203.0 (177.0-241.0)	243.5 (205.2-288.8)	0.6 (0.5, 0.7)	<0.001
PT	11.9 (1.5)	12.3 (2.1)	0.3 (0.1, 0.4)	<0.001
AST	24.0 (19.0-30.0)	29.0 (23.0-41.0)	0.4 (0.3, 0.5)	<0.001
LYMPH	10.4 (6.3-15.9)	7.0 (4.6-11.7)	0.3 (0.2, 0.4)	<0.001
Chlorine	103.2 (4.6)	102.0 (5.6)	0.2 (0.1, 0.4)	<0.001
Potassium	3.7 (0.5)	3.7 (0.6)	0.1 (-0.0, 0.2)	0.024
RBCDW	14.3 (13.3-41.5)	15.9 (13.9-44.2)	0.3 (0.2, 0.4)	<0.001
UA	292.8 (215.2-381.0)	354.2 (278.3-425.6)	0.4 (0.3, 0.5)	<0.001
AA	1.2 (0.9-1.6)	1.4 (1.1-1.8)	0.4 (0.3, 0.5)	<0.001
Phosphorus	0.9 (0.8-1.1)	0.8 (0.7-1.1)	0.0 (-0.1, 0.2)	0.008

Data were presented as mean (SD) or median (interquartile range). AA, the ratio of alanine aminotransferase to AST; AST, aspartate aminotransferase; CREA, creatinine; GLU, blood glucose; LDH, low-density lipoprotein; LYMPH, percentage of lymphocytes; PT, prothrombin time; RBCDW, red blood cell distribution width (CV); UA, uric acid; WBC, white blood cell count.

Supplementary Table S4. Distribution of extracted laboratory features with regard to the data sets.

Variable	Development set (n=1405)	Test set (n=430)	Standardize Difference* (95% CI)	*P*-value
GLU	7.2 (6.0-9.1)	7.4 (6.2-10.0)	0.1 (0.0, 0.2)	0.046
CREA	78.0 (64.0-97.9)	73.0 (60.0-97.0)	0.0 (-0.1, 0.2)	0.011
WBC	11.2 (4.5)	11.1 (4.2)	0.0 (-0.1, 0.1)	0.737
LDH	207.0 (179.0-247.2)	215.0 (183.0-261.0)	0.1 (-0.0, 0.2)	0.062
PT	11.6 (11.0-12.4)	11.7 (11.2-12.6)	0.1 (0.0, 0.2)	0.009
AST	25.0 (20.0-32.0)	24.0 (20.0-32.0)	0.1 (-0.1, 0.2)	0.465
LYMPH	9.9 (5.9-15.1)	9.2 (5.8-15.1)	0.0 (-0.1, 0.2)	0.433
Chlorine	103.2 (4.7)	102.3 (5.0)	0.2 (0.1, 0.3)	<0.001
Potassium	3.7 (0.5)	3.7 (0.5)	0.0 (-0.1, 0.1)	0.821
RBCDW	14.5 (13.4-41.9)	14.4 (13.3-42.4)	0.0 (-0.1, 0.2)	0.957
UA	304.1 (215.1-392.7)	310.0 (245.1-385.2)	0.1 (-0.0, 0.2)	0.118
AA	1.3 (0.9-1.6)	1.2 (0.9-1.6)	0.1 (-0.1, 0.2)	0.372
Phosphorus	0.9 (0.7-1.1)	0.9 (0.7-1.0)	0.1 (-0.0, 0.2)	0.292

Data are presented as mean (SD) or median (interquartile range). AA, ratio of alanine aminotransferase to AST; AST, aspartate aminotransferase; CREA, creatinine; GLU, blood glucose; LDH, low density lipoprotein; LYMPH, percentage of lymphocytes; PT, prothrombin time; RBCDW, red blood cell distribution width (CV); UA, uric acid; WBC, white blood cell count.
